# Intradural Disc Herniation: A Case Report and Literature Review

**DOI:** 10.7759/cureus.7600

**Published:** 2020-04-09

**Authors:** Almas Ashraf, Zaheer-ud-Din Babar

**Affiliations:** 1 Neurosurgery, Shifa College of Medicine, Islamabad, PAK; 2 Neurosurgery, Shifa International Hospital, Islamabad, PAK

**Keywords:** lumbosacral region, neurosurgery, magnetic resonance imaging

## Abstract

Intradural disc herniation is a rare complication that is difficult to diagnose preoperatively, despite the availability of various radiological imaging tools. We report a case of a 61-year-old man with L4-L5 lumbar disc herniation who presented with back pain radiating to both legs, difficulty in walking, and urinary incontinence. Magnetic resonance imaging showed a disc bulge at the L4-L5 level. However, fragment was not seen until perioperatively; a disc fragment was found in the intradural space. While various radiological techniques have been reported in the literature for diagnosing herniated discs, an absolute diagnosis of intradural disc herniation by a single radiological investigation is unreliable. The current case demonstrates the limitations of various diagnostic methods available. We also present a review of the literature regarding possible modalities to aid diagnosis.

## Introduction

An intradural disc herniation (IDH) is defined as a nucleus pulposus fragment of the intervertebral disc intruding through the dural sheath into the thecal sheath. It is a rare complication of disc herniation with an incidence rate of about 0.27% to 0.33% of all cases of disc herniation and peak incidence in the fifth and sixth decades of life [1]. The ﬁrst report of a lumbar IDH was by Dandy in 1942 [2]. Approximately 92% of IDHs occur in the lumbar region, as in our case, and most commonly affect the L4-L5 region (55%), followed by the L3-L4 region (16%), and the L5-S1 region (10%) [2].

## Case presentation

A 61-year-old man with diabetes and hypertension presented to the hospital complaining of chronic back pain for the past four years. The pain was radiating down both legs all the way to his feet. This pain worsened recently with the addition of a loss of urinary control for the prior month. The pain increases when the patient coughed or sneezed. He has difficulty climbing stairs and getting up from low seats. He is unable to walk without support. He also has nocturnal pain. However, he gave no history of numbness, tingling sensation, or weakness from levels L3-S2.

**Figure 1:**
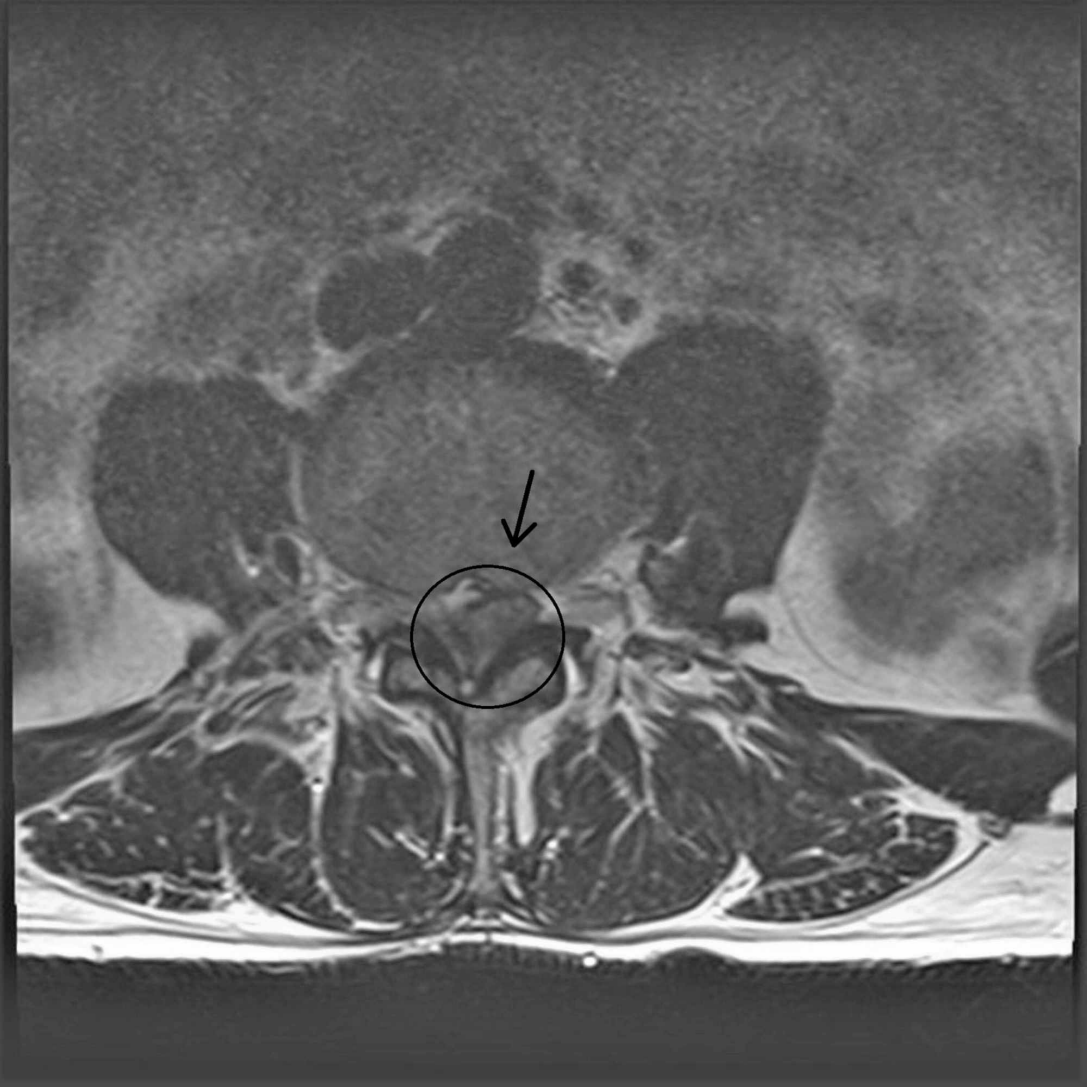
Axial image of MRI that shows disc fragment at the L4-L5 level.

On examination, the patient could not walk without support. The straight leg raising test revealed pain at 60 degrees bilaterally. Power was rated at 3/5 in his left leg and 4/5 in his right leg of knee flexion, knee extension, foot dorsiflexion, and plantar flexion. Perineal sensations were lost, while tingling sensation were present at posterior aspect of leg and feet. His knee reflexes were intact at grade 2+, but his ankle reflex was absent in the left leg that is grade 0 and decreased in the right leg at grade 1+. X-rays showed an osteoporotic spine only. Magnetic resonance imaging (MRI) revealed a large disc bulge at the L4-L5 level with effacement of the nerve root on the left side as seen in Figure 1.

L4/L5 decompression surgery was planned. Intraoperatively, an L4 laminectomy was performed, and the patient was found to have severe bilateral compression of nerve roots at the L4-L5 level. The dural disc was incised. Furthermore, an intradural L4-L5 disc fragment was incidentally found as felt externally and then removed. The dura was closed with primary closure and ensured the nerve root was decompressed. Postoperatively, the patient recovered with no complications.

## Discussion

The exact etiology of IDH is unknown, but theories are proposed in the literature. The migration of a disc nucleus pulposus to the intradural site requires perforation of the dura matter, the annulus fibrosis, and posterior longitudinal ligament. This may be caused by inflammation and adhesions due to degenerative disc disease, or it may be a result of postoperative iatrogenic procedures. Other causes include congenital narrowing of the spinal canal, fineness of the dura mater, congenital thinness of the dura mater or fusion of the dura mater, and posterior longitudinal ligament [2].

A preoperative diagnosis of IDH is difficult. Lesions might be ignored or mistaken for other findings on imaging. In almost all cases described in the literature, a definite diagnosis was made only on surgery, not via imaging along. The MRI in our case did not indicate the presence of intradural disc fragments.

Hidalgo-Ovejero et al. reported the finding of epidural gas in a computed tomography (CT) scan as a diagnostic marker for IDH [3]. The gas is 90% nitrogen and carbon dioxide and can migrate to the epidural space with fragments and can be present in pseudocysts or originate in adjacent structures [4]. Sometimes this air is noted as sequestrum in the intradural space or spinal canal on CT images.

Some MRI findings for intradural lumbar disc herniation might aid in preoperative identification. Lidov et al. reported the case of a 53-year-old patient in whom MRI was able to reveal both the intra- and extradural parts of the lesion [5]. The MRI highlighted a large migrated fragment but also revealed irregular enhancement along the intradural portion of the hernia and enhancement along the spinal roots of the cauda. Sasaji et al. described a “Y-sign” in case of intradural extra-arachnoid disc herniation. As the arachnoid was peeled from the dura, it appeared as two lines, then one line of the dura and the arachnoid [6].

Floeth and and Herdmann considered the possibility to differentiate between intraspinal/intradural sequester and an intraspinal/intradural tumorous or cystic space-occupying lesion through high-resolution MRI techniques [7]. Choi et al. reported two signs of MRI: the loss of posterior longitudinal ligament continuity and a “hawk-beak-sign” on a T2-weighted image [8].

Wasserstrom et al. made a preoperative diagnosis through gadolinium-enhanced MRI. They notice a ring enhancement in a T1-weighted image at L4-L5 after injection of contrast medium, produced by granulation tissue around the lesion [9,10]. Hida et al. also described a 62-year-old man in whom an L2-L3 intradural herniation diagnosis was made based on a T1-weighted MRI showing an isodense intradural mass and revealed the typical ring enhancement with gadolinium [10]. The radiologic diagnosis of intradural herniation is possible in carefully selected patients using MRI with gadolinium.

## Conclusions

We presented a case of 61-year-old male who had back pain, loss of urinary control, and difficulty in walking. His MRI showed a large disc fragment in the region of L4-L5. However, intraoperatively, the fragment was found intradurally. Therefore, the exact diagnosis of IDH was not clear until during the surgery. Preoperative markers for such cases described in the literature are detection of epidural gas in a CT scan, or air noted in the intradural space on CT. Moreover, MRI might rarely shows it as migrated fragment or irregular enhancement or the characterized "Y-sign" or “hawk-beak-sign.” Preoperative diagnosis of IDH is difficult, although gadolinium-enhanced MRI may be useful for diagnosis. The selection criterion to determine good candidates for radiology techniques is needed, which would also minimize the number of different imaging procedures performed for each patient and impart a high degree of sensitivity and specificity for diagnosing it preoperatively.
